# High rates of benzimidazole-resistance-associated alleles in *Haemonchus contortus* and detection of resistance against macrocyclic lactones in strongylids from German alpaca herds

**DOI:** 10.1186/s13071-024-06377-4

**Published:** 2024-07-09

**Authors:** Barbara Hinney, Sandra Wiedermann, Xenia Vaneev, Katharina Muhm, Anja Joachim, Thomas Wittek

**Affiliations:** 1 Department of Pathobiology, Institute of Parasitology, University of Veterinary Medicine Vienna, Vienna, Austria; 2 Department for Farm Animals and Veterinary Public Health, University Clinic for Ruminants, University of Veterinary Medicine Vienna, Vienna, Austria

**Keywords:** Anthelmintic efficacy, Anthelmintic resistance, Nematodes, dPCR, Benzimidazoles, Moxidectin, Monepantel, Ivermectin

## Abstract

The population of South American camelids (SAC) has been steadily growing in Europe, where they are confronted with the regional endoparasite population of ruminants. As there are no anthelmintic drugs registered for use against nematode infections in SACs, anthelmintics (AH) available for ruminants or horses are usually applied. Reports indicating potential failures in administered AH are increasing. However, the generally low egg counts in SACs complicate the application of resistance tests in the field. The present study reports a follow-up study on SAC farms where anthelmintic resistance (AR) was suspected. The aims were (i) to repeat faecal egg count reduction tests (FECRTs) on potentially affected farms identified in a previous study with larger sample sizes, (ii) to verify suspected AR of *Haemonchus contortus* against benzimidazoles (BZ) by performing a single-nucleotide polymorphism (SNP) analysis using digital polymerase chain reaction (dPCR), and (iii) to apply the mini-FLOTAC technique for more reliable results at low egg counts in line with current recommendations. Seven farms (9–46 animals each) were examined by coproscopy, larval differentiation and SNP analysis. A FECRT was performed on six of these farms with moxidectin (three farms), monepantel (two farms) and ivermectin (one farm). The FEC was calculated according to the current World Association for the Advancement of Veterinary Parasitology (WAAVP) guidelines with the clinical protocol (a newly introduced variant of FECRT which can be used for smaller sample sizes and lower egg counts on the cost of sensitivity) and an expected efficacy of 99%. A high level (> 90%) of BZ-resistance-associated SNPs on codon 200 of *H. contortus* was observed on all farms. With the FECRT, resistance was demonstrated for ivermectin (74% FECR), while it remained inconclusive for one farm for moxidectin treatment. Sustained efficacy was demonstrated for the remaining treatments. This study showed an advanced level of BZ resistance in *H. contortus* of SACs and the development of AR against macrocyclic lactones on some farms. Thus, constant monitoring of AH treatment and sustainable worm control methods both need to be applied.

**Graphical Abstract:**

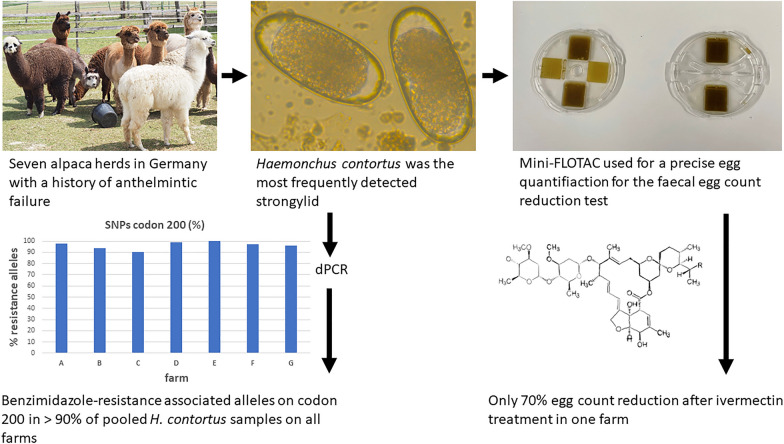

**Supplementary Information:**

The online version contains supplementary material available at 10.1186/s13071-024-06377-4.

Infections with gastrointestinal nematodes are a major health concern in South American camelids (SAC), causing production losses and clinical disease with variable severity [1]. These animals may share several helminths with sheep, goats and cattle, and co-grazing with ruminants is a risk factor for the establishment of high endoparasite burdens in camelid herds [1]. Conversely, alpacas could also serve as a reservoir for the trichostrongylid *Haemonchus contortus* for ruminant infections [2]. *Haemonchus contortus* is one of the most pathogenic parasites in both groups of hosts. High burdens can lead to severe disease with anaemia, hypoproteinaemia, apathy and weight loss, and can even result in death [3]. Anthelmintics are a key tool to manage helminth infections; however, the emergence of anthelmintic resistance (AR) threatens livestock farming worldwide. Reduced anthelmintic efficacy indicating resistance is also increasingly reported in SACs [4–8], and *H. contortus* in particular is prone to fast AR development under treatment [9]. There are no anthelmintic drugs registered for use in SACs, and several studies indicate that dosages previously used for benzimidazoles (BZ) and macrocyclic lactones (ML) are below the effective doses for these species [1, 5, 10, 11]. Also, some application routes such as pour-on are inadequate to reach effective doses in SACs [10]. Underdosing is a major risk factor for the development of AR, and therefore anthelmintic efficacy should regularly be monitored [1, 12]. Of all methods that are available to determine AR, the faecal egg count reduction test (FECRT) is most frequently applied. It is applicable to all available classes of anthelmintics, but it provides low sensitivity [13]. In addition, SACs often have low egg counts [14], which often excludes a number of algorithms for FECR calculation (e.g. Coles et al. [13]). A further challenge encountered with FECRTs in SACs is that for most anthelmintics, the effective doses are unknown, which impedes interpretation. This also applies to BZ used in SACs. For BZ, however, highly sensitive molecular tests are available to detect resistance-associated single-nucleotide polymorphisms (SNPs) in strongylid populations [13]. SNPs on codon 200 of the ß-tubulin gene are frequently associated with BZ resistance, while in some geographical regions, SNPs on codons 167 and 198 are predominant [15]. Currently, no molecular tests for AR determination are available for ML or monepantel.

Sustainable parasite control strategies are applied to slow down the development of AR in livestock [16, 17]. One of these strategies is the targeted selective treatment (TST) approach where only selected animals are treated with anthelmintics while a proportion of animals remain untreated to contribute to the refugium of susceptible worms [18]. Overdispersion of parasite egg shedding is a biological feature of nematode infections and provides the basis for selection of animals in TST based on FEC [18, 19].

In a previous study, we examined 27 alpaca herds in Germany and monitored the efficacy of anthelmintic treatment by FECRT [14]. The results indicated wide variations in efficacy of BZ and considerable levels of AR (suspected in 72% of the 13 farms treated with BZ). Also, monepantel and moxidectin were not sufficiently effective on two and six farms (40% and 100% of those receiving the respective drug).

To characterize egg shedding within selected herds and to characterize lack of treatment efficacy in detail, farms with previously suspected resistance were included in the present follow-up study. In addition, we wanted to further investigate the extent of BZ resistance by performing SNP analysis to detect the frequency of BZ-resistance alleles of *H. contortus*. As the SNP analysis is a molecular test independent of anthelmintic treatment, this test was well suited for analysing AR of *H. contortus* against a compound where the initial efficacious dosage for alpacas is unknown. Another aim was to perform FECRTs with moxidectin or monepantel where the effective dosage for alpacas had previously been determined [11, 20], and we used mini-FLOTAC, which has higher accuracy than the McMaster technique [17, 21], to overcome the problem of the generally low egg counts in SACs. The FECRT was conducted within a TST approach where only high egg shedders or animals that where otherwise considered to profit from anthelmintic treatment were dewormed.

Seven farms (A–G) volunteered for this follow-up study. Between May and September 2020, a total of 224 fresh individual faecal samples (Table 1) were collected by the farmers and sent to the institute by courier service, where they were examined within 36 h by Mini-FLOTAC according to the protocol for herbivores with a lower detection limit of five eggs per gram of faeces (EpG) [21]. Strongylid eggs were counted, while other worm eggs and coccidian oocysts were documented qualitatively.

**Table 1:** Characterization of strongylid egg shedding on the farms

Farm	*n*	Mean EpG (SD)	Minimum EpG	Maximum EpG	Median EpG	Aggregation factor (*ĸ*)
A	23	249 (548)	< 5	2685	60	0.42
B	35	138 (48)	< 5	2680	15	0.25
C	30	85 (85)	< 5	1750	5	0.15
D	46	140 (320)	< 5	2050	47.5	0.5
E	40	71 (147)	< 5	865	15	0.39
F	9	80 (159)	< 5	525	20	0.43
G	41	65 (118)	< 5	580	25	0.59

A sedimentation examination for liver fluke egg detection was performed. Samples positive for strongylid eggs were pooled per farm and subjected to coproculture for subsequent larval differentiation. Besides farm F where no FECRT was performed, no larvae or insufficient larvae post-treatment were available on farms A, E and G, since drug efficacy was nearly or exactly 100%. Larvae were further examined by a digital polymerase chain reaction (dPCR) assay for detection of BZ-resistance-associated SNPs in* H. contortus* on codons 200, 167 and 198 (methods described in [22]).

The minimum egg count (strongylid eggs) for the treatment of animals was 200 EpG. Additionally, animals with lower egg counts that were considered to benefit from treatment (e.g. in cases of poor body condition, anaemia or signs of indigestion) by the farmer and/or attending veterinarian were included, leading to 8–12 animals per treatment group (Table 2).

**Table 2:** Results of FECRT on the farms from the previous and present study as well as results of SNP analysis

Farms	Results from Kultscher et al. [14]			Results from the present study				
	FECR% (95% CI) [interpretation^a^]; *n*			Mean EpG before treatment	Drug [interpretation^b^]	FECR% (90% CI); *n*	SNP codon 200 in % (95% CI)	SNP codon 167 in % (95% CI)
	FBZ	MOX	MON					
A	15 (0–35) [R]; 11	100 (94–100) [S]; 5	–	591	MOX [S]	99.8 (99.5–100); 9	97.86 (97.64–98.06)	2.23 (2.03–2.45)
B	26 (0–50) [R]; 8	30 (0–59) [R]; 3	–	383	MOX [I]^c^	94.5 (86.7–99); 12	93.86 (92.91–94.69)	< 5
C	47 (33–60) [R]; 16	100 (96–100) [S]; 5	–	247	IVM [R]	74.6 (34.9–96.8); 10	90.27 (89.22–91.23)	5.34 (4.82–5.91)
D	74 (40–90) [R]; 6	–	73 (43–88) [R]; 8	440	MOX [S]	98.1 (96.2–99.4); 11	98.97 (98.30–99.38)	< 11
E	45 (7–66) [R]; 3	99 (81–100) [SR]; 2	99 (87–100) [SR]; 7	203	MON [S]	99.7 (99.4–100); 12	100	< 9
F	7 (0–58) [R]; 2	97 (53–100) [SR]; 2	–	–	Not performed		97.05 (96.48–97-53)	< 4
G	61 (41–76) [R]; 13	93 (91–95) [SR]; 15	99 (93–1) [S]; 12	216	MON [S]^d^	97.4 (91.9–99.9); 8	95.58 (93.00–97.23)	< 36

*R* resistance, *S* susceptibility, *I* inconclusive, *SR* suspected resistance, *CI* confidence interval, *FBZ* fenbendazole, *MOX* moxidectin, *MON* monepantel, *IVM* ivermectin; < *x*% = below background level (see supplementary file)

^a^Calculated with egg counts, 95% CI; definition of AR according to [13]

^b^Calculated with fecrt.com, 90% CI; definition of AR according to [23], clinical protocol, grey zone 90–99%; CI calculated with the delta method [26]

^c^Resistance according to the beta-negative binomial BNB [25] method and CI calculated according to [24]; inconclusive was the preferred interpretation by the programme

^d^Inconclusive according to the BNB [25] method and CI calculated according to [24]; inconclusive was the preferred interpretation by the programme

Including animals with an egg count below 200 in the FECRT is in line with the current World Association for the Advancement of Veterinary Parasitology (WAAVP) guidelines, where the total number of eggs counted are relevant for the statistical power of FECRT [23]. The software provided via https://www.fecrt.com/ was used to determine the appropriate FECRT protocol, the aggregation factor (*ĸ*) for the egg shedding within the whole herd by a negative binomial distribution and the confidence intervals for the FECRT (based on the delta method) [24, 25]. The rate of FECR was calculated with the standard formula [100% × (1 − mean post-treatment FEC/mean pre-treatment FEC)] [23].

Definition of AR was based on Kaplan et al. [23], where *susceptibility* is present when the lower 90% CI is greater than or equal to the lower efficacy threshold and the upper 90% CI is greater than or equal to the expected efficacy. *Resistance* is present when the upper 90% CI is less than the expected efficacy, and results are *inconclusive* if neither of the above given criteria is met.

The current guidelines include both a clinical protocol and a research protocol [23]. The clinical protocol is designed for practical use with lower numbers of animals and/or counted eggs than required for the research protocol. Both protocols lead to statistically meaningful results, but the clinical protocol may not detect emerging resistance as effectively as the research protocol, leading to more frequent instances of inconclusive outcomes [23]. For all farms, the sample size was sufficient for the clinical but not for the research protocol. We adopted the expected efficacy for cattle with a grey zone of 90–99% [23].

It can be discussed whether an expected efficacy of 99% for anthelmintics against ruminant nematodes should be applied to the interpretation of anthelmintic efficacy in alpacas. However, in a previous study, monepantel (7.5 mg/kg orally [p.o.]) demonstrated 100% efficacy in llamas [11]. The interpretation of moxidectin efficacy presents a more complex challenge. Following a treatment failure of 0.2 mg/kg p.o. moxidectin in SACs [7], a higher dose of 0.4 mg/kg p.o. (also used in the present study) was recommended [20]. This was based on a dose titration that was performed a decade after moxidectin was marketed for livestock in the USA. In alpacas, the highest efficacy achieved was 98%, while it reached 100% on some llama farms [20]. Thus, the initially effective dose in alpacas remains unclear, potentially leading to an overestimation of the expected efficacy of moxidectin if the upper margin is set at 99%. We, however, retained this margin since the current WAAVP guidelines also set the expected efficacy for goats at 99%, a species where there is a similar lack of knowledge on the originally effective dose.

Selection of the drug to be applied was based on the decision of the farmer and attending veterinarian (Table 2). On farm F, only one animal qualified for a FECRT, and this farm was thus excluded from this test. Animals treated with moxidectin received 0.4 mg/kg p.o. [20], and animals treated with monepantel received 7.5 mg/kg p.o. [11]. One farm encountered a *Psoroptes* sp. infestation in the alpacas, and the veterinarian decided to treat all animals with ivermectin subcutaneous injection (s.c.) at a dosage of 20 mg/animal which, depending on the weight of the animals, corresponded to a dosage of 0.2–0.4 mg/kg ivermectin. Treatment was not performed immediately but 2 to 22 days after sampling (except for the farm with ivermectin treatment, where animals were treated on the day of sampling). Individual faecal samples were taken 14 days post-treatment (15–17 days on farms treating with moxidectin) and examined with Mini-FLOTAC.

Upon initial examination, strongylids were detected on all farms and in 82% of the animals, *Nematodirus* spp. on all farms and 24% of the animals, *Capillaria* spp. on four farms and 9% of the animals, and *Eimeria* spp. on all farms and 61% of the animals (Supplementary file 1). Mean EpG levels of strongylids were generally low (see Table 1), as previously observed in alpacas [14, 26]. The low FEC can probably be attributed to the farm management, as faeces were removed daily by the farmers, pastures were not overgrazed and none of the farms practised co-grazing with sheep. Overdispersion could be observed on all farms with *ĸ*-values between 0.12 and 0.59 (Table 1) and 10% of animals contributing to the majority of egg shedding (Fig. 1).

**Fig. 1:**
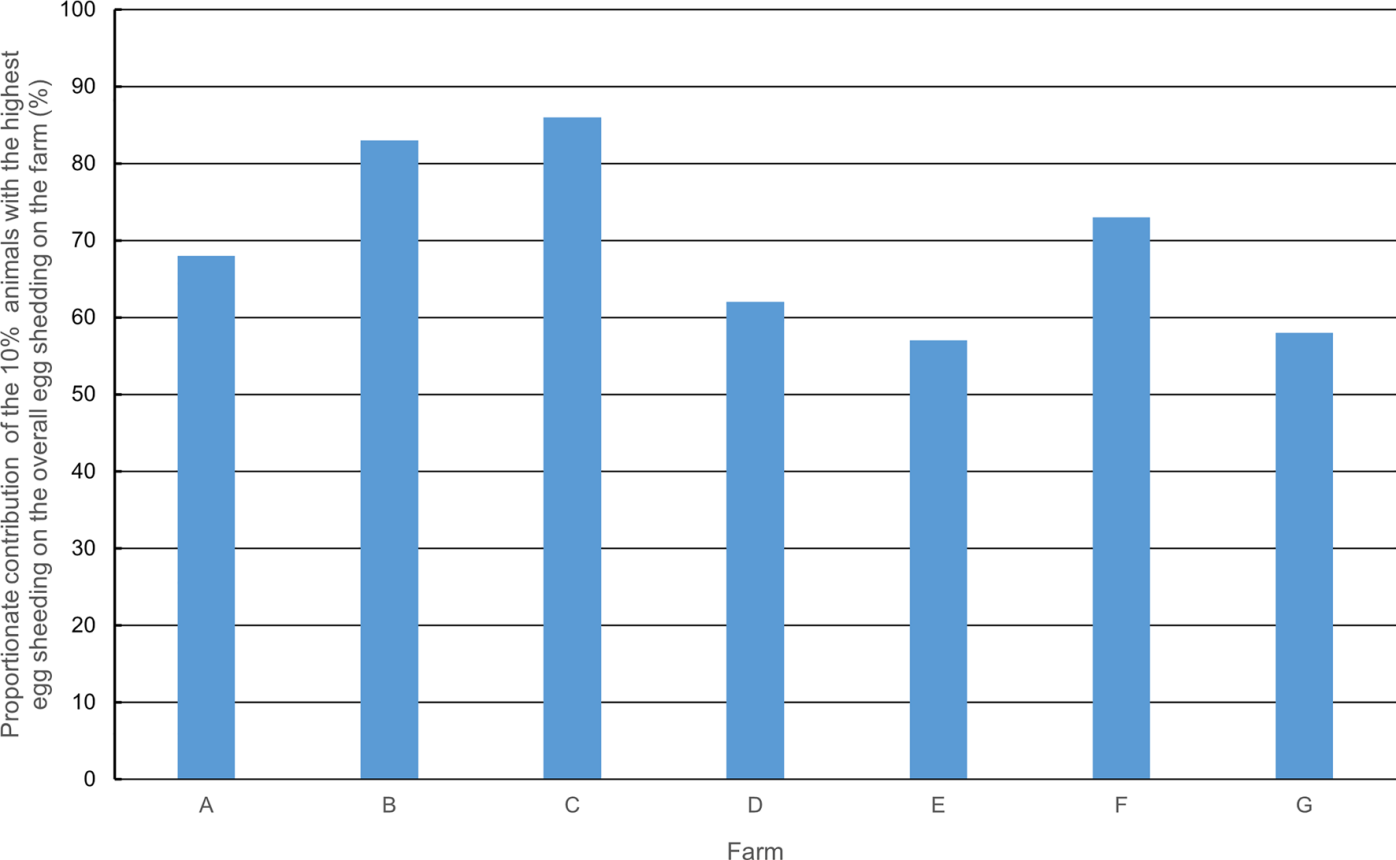
Overdispersion of strongylid egg shedding. The 10% of animals with the highest egg shedding contributed to the majority of overall egg shedding of the herd

Thus, in all herds a TST approach based on FEC was feasible. In the present study, the cut-off for treating animals was set at an EpG ≥ 200. This was a rather conservative approach, as in farms with a high proportion of *H. contortus*, obvious clinical signs were mostly observed in animals with an EpG of 1000 or above [3]. More research is needed to clearly determine the EpG cut-off value for treatment in alpacas. Ideally, for selection of animals for treatment, decisions should be based not only on egg counts but also in combination with physical examination. In particular, body condition scoring and, on farms with a predominance of *H. contortus*, FAMACHA^©^ scoring was shown to be an adequate technique to select animals that would benefit from treatment [3].

Larval cultures revealed a predominance of *Haemonchus* spp. on all farms (53–97%) before treatment, followed by *Cooperia* spp. and *Trichostrongylus* spp. (Fig. 2). Thus, selection of animals for TST based on the FAMACHA^©^ system would be an option on these farms.

**Fig. 2:**
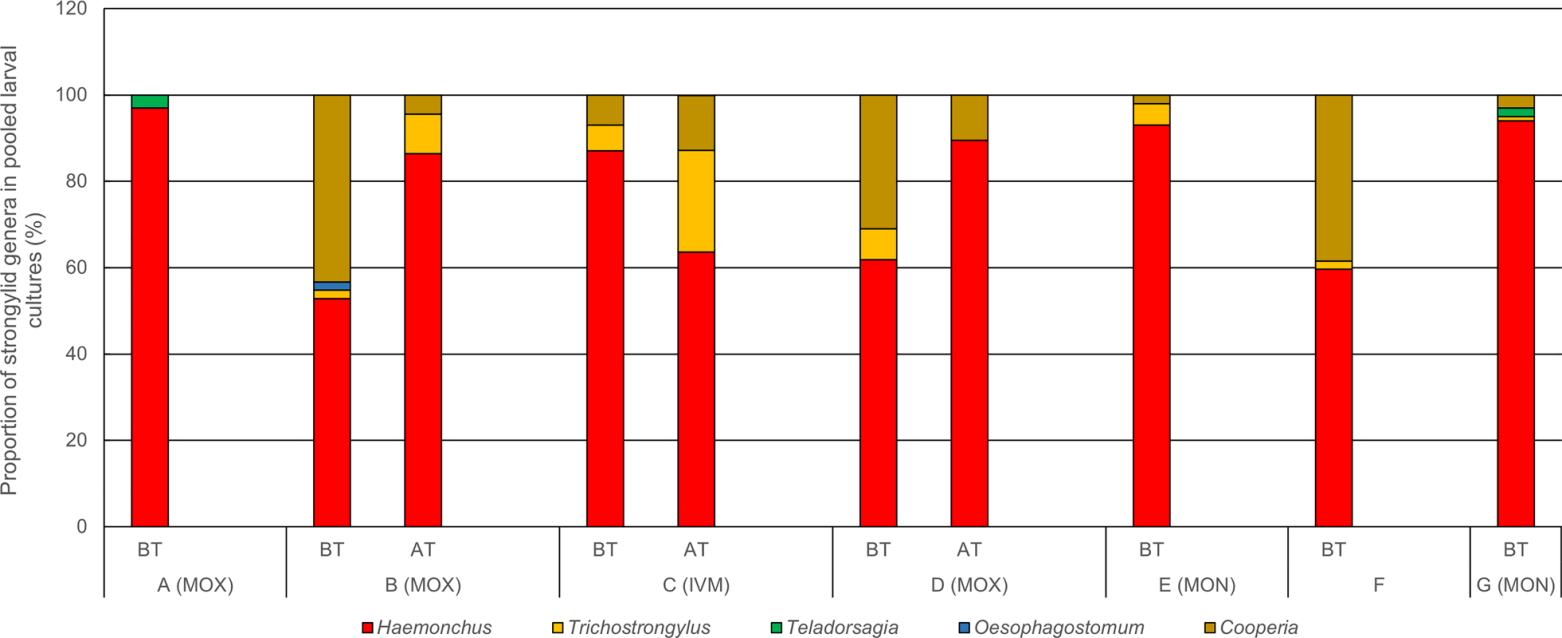
Relative composition of trichostrongylid genera and the genus *Oesophagostomum* before (BT) and after treatment (AT) in farms A–G. *MOX* moxidectin, *IVM* ivermectin, *MON* monepantel

In the pooled samples, *H. contortus* resistance alleles on codon 200 showed very high allele frequency (Table 2). Resistance alleles on codon 167 were rarely observed, and mutations on codon 198 were not detected. The high level of resistance alleles in codon 200, frequently observed in *H. contortus* from sheep in central, western and northern Europe [15, 27–29], also occurred in *H. contortus* from alpacas in the present study. It can therefore be assumed that the previously observed treatment failure of BZ on these farms in 2018 [14] could likely be attributed to AR and not (or not exclusively) to an insufficient dose.

The FECRT% indicated susceptibility on both farms with monepantel treatment. It thus did not confirm the suspected resistance observed in farm E from the previous study [14]. On the three farms where treatment with moxidectin was performed, FECRT indicated susceptibility two times, on farms A and D and inconclusive results once on farm B. Consequently, the previously observed suspected resistance on farm B could not be confirmed, although the repeated observation of inconclusive results indicates that the selection for AR has occurred, albeit in an early stage. The farmer reported that she strongly adhered to a selective treatment programme, and it can be assumed that this is one of the reasons why AR of moxidectin had not progressed further since 2018. Resistance was detected on the farm with ivermectin treatment (Table 2). It can thus be assumed that monepantel is still effective on the examined farms, while selection for resistance might be in progress on one of the farms with moxidectin treatment. Resistance against ivermectin is very likely already far progressed on the farm in question.

However, FECRT results should be interpreted with care here, as the low FECs together with a sample below the required sample size for the research protocol possibly compromise the validity of the FECRT. However, it is mentioned in the WAAVP guidelines that the clinical protocol is as robust as the research protocol but more often leads to inconclusive results, which also was the case in one of the examined farms in the present study. Additionally, we used the Mini-FLOTAC technique that provides high accuracy, which increases the validity of the results [23].

The low efficacy of the ivermectin treatment could also be attributed to the administration route. Generally, poor efficacy of injectable products compared to oral administration is observed, and oral anthelmintic administration is considered to be the most effective technique for endoparasite treatment in livestock [30]. However, there were contradictory results in studies in SACs looking at plasma concentrations of moxidectin and ivermectin with either oral or parenteral (s.c.) treatment, so the ideal administration route for ivermectin in these hosts is unclear [12]. Injection of ivermectin is recommended for psoroptic mange treatment [10]. However, this might also select for resistant endoparasites as an off-target effect of the treatment. We therefore recommend that a FEC also be performed after ectoparasite treatments with an endectocide. If nematode eggs are counted, treatment with another compound to remove resistant worms should be performed. A similar concept is applied by composite treatment practiced in Australia and the USA, which is considered a suitable strategy to prevent the spread of resistant worms [4].

Strongylids shed after treatment can be considered less susceptible to the anthelmintic drug than expected and, generally, care should be taken that a refugium for susceptible worms is provided when anthelmintic treatment is applied.

Data on the relative proportion of the different strongylid genera post-treatment were available for three farms. The proportion of *Haemonchus* increased on two farms with moxidectin treatment, while it decreased in favour of *Trichostrongylus* on the farm with ivermectin treatment (Fig. 2). The predominant worm genus conferring AR on the examined farms was thus *Haemonchus*, probably followed by *Trichostrongylus*. It should be noted that there are limited keys available for the differentiation of third-stage larvae (L3) of the genus *Camelostrongylus*. Additionally, *Camelostrongylus* L3 seem to be hardly distinguishable from *Teladorsagia* [31]. However, in the present study, L3 were classified as *Teladorsagia* spp., since *Camelostrongylus* spp. had not yet been described in Central Europe.

In conclusion, AR already appears to be advanced in alpaca herds in Germany. Thus, treatment with BZ should not be recommended without confirmed efficacy. Efficacy monitoring is generally strongly recommended after any anthelminthic treatment, and the clinical protocol recommended by the new WAAVP guidelines also allows testing for efficacy if a lower number of animals in a herd are treated within a TST approach. Alternative strategies apart from application of anthelmintics (e.g. pasture management) should be considered, and all strategies that maintain a refugium for susceptible worms should be encouraged. Given the fact that overdispersion and low egg counts occur in most alpaca herds, TST strategies would be practicable and should be applied.

### Supplementary Information

Supplementary Material 1.

## Data Availability

All data generated or analysed during this study are included in this article and its supplementary material.
